# Report of the Meeting of the Western Dental Society at Chicago, Ill., on July 30th, 1856

**Published:** 1856-10

**Authors:** Solyman Brown


### SELECTED ARTICLES.

### ARTICLE III.

*Report of the Meeting of the Western Dental Society at Chicago, Ill., on July 30th,* 1856.

PRESENT.—Drs. Allport, I. C. Quinlan, W. H. Kennicott, E. Honsinger, T. P. Abell, E. A. Bogue, Chicago, Ill.; J. S. Clark, S. F. Knapp, New Orleans; C. W. Spaulding, A. Blake, Henry Barron, S. Dunham, H. J. B. McKellops, St. Louis; H. N. Lewis, Quincy, Ill.; A. M. Kelsey, Geneva, Ill.; D. W. Perkins, Rome, N. Y.; H. R. Smith, Terre Haute, Ind.; C. P. Fitch, Hanson, Milwaukie; I. P. Norman, Bockford, Ill.; Anderson, Hannibal, Mo.; Solyman Brown, New York; William Smith, Ottawa, Ill.; A. T. Metcalf, Kalamazoo, Mich.; Mansfield, Niles, Mich.; E. J. E. Carpenter, Joliet; C. J. Reynolds, Dixon, Ill.; A. Gibbs, Chicago, Ill.

In the absence of the President, D. E. Hale, of St. Louis, Dr. Allport, one of the Vice-Presidents was called to the chair.

Dr. Allport said, that in the unexpected absence of the President, he should detain the meeting with but a very few remarks.

A few years had effected a remarkable change in the dental profession. Formerly a knowledge of its more elevated practice was confined to the larger cities. Now the science of dentistry was extended over the whole country, north, south, east and west. This was the normal result of a generous liberality on the part of a few eminent practicioners, who had made known the principles and practice of dentistry to their professional brethren, as well as to students in the art, both privately and by means of periodicals and colleges, established expressly for the purpose, in various parts of the country. The names of

Parmly, Harris, Maynard, Taylor, Townsend, Dwinelle, Solyman Brown, Dunning, Westcott and others, would readily occur in this connection.

In pursuance of the same object, and in imitation of these noble examples, we organized this Society last April, in the Great West, and have new met for mutual instruction and improvement.

It was then, on motion, *Resolved*, that Dr. Solyman Brown, of N. Y., Drs. Clarke and Knapp, N. O., Dr. Perkins, of Rome, N. Y.; and Dr. Smith, of Terre Haute, Ind., (one of the Professors of the Cincinnati College of Dental Surgeons,) be invited to participate in the business of the present meeting.

After reading and approving the minutes of the last meeting, a Committee consisting of Drs. Blake, Dunham and Clark reported as the special business of the session, the following subjects for discussion, viz. The Extraction of the deciduous teeth of children; The Plugging of the fangs of teeth; The use of Gutta-Percha as the basis of artificial teeth; and Crystal Gold.

This judicious selection of subjects was approved by the meeting.

Dr. Clarke opened the discussion on deciduous teeth. He remarked that the subject was one which had been too much neglected by the profession, and yet was one of pre-eminent importance. If deciduous teeth are removed as a general rule, it ought to be by the absorption of the roots in proportion as the permanent teeth advance to supply their place. The use of the first set is to prepare the way for their successors, and to perform the uses of the second set of teeth until the gums are fully developed to receive them.

The wholesale extraction of deciduous teeth, for the purpose of gratifying nurses who dislike the crying of children, or parents who are ignorant of the uses of these organs beyond the mere mastication of food, is a very bad practice. He does not extract the tenth part of those which are presented to him for that purpose.

These teeth preserve the form of the jaws, and unless they become loose by absorption of the roots, and no longer serve that purpose, as a general rule should never be removed. Nature generally removes them in her own right time, and by means altogether preferable to the forceps of the dentist.

Dr. Kennicott inquired of Dr. Clarke what had been his successes in stopping deciduous teeth.

Dr. Clarke responded that he had never courted success in that line. He called it only *stuffing.*

Dr. Perkins remarked, that the development of the human physical system was harmonious throughout, and simultaneous in all its parts. He described the development of the two successive sets of human teeth. In treating the teeth of children he always regards primarily the welfare of the adult teeth which succeed them. The teeth of children are now developed much earlier in this country than in former years, the probable result of the different habits of society at successive periods.

Dr. Knapp inquired of Dr. Perkins whether he would extract deciduous teeth, when they were decayed to the nerve; and was answered that it would depend somewhat upon circumstances.

Dr. Knapp remarked, that children should be taught to exercise their teeth in the mastication of hard food, which is necessary to their healthy development.

Dr. Clarke remarked that Dr. Knapp had been trying the experiment for four or five years, of brushing his children's teeth morning and evening, with his own hands, not being willing to entrust the experiment to nurses, and that his success in preserving them seemed thus far very perfect.

Dr. Perkins does not allow his children to use vinegar; in consequence of the deleterious effects of all acids except those of the milder forms, such as found in fruits. He does this by way of experiment, as he thinks vinegar injures the tender enamel of children's teeth.

Dr. Dunham inquired whether there is any particular advantage in filling children's teeth, to which Dr. Clarke replied in the negative.

Dr. Allport stated that he had been in the habit of doing so, when it seemed proper. When practicable he files instead of filling. He thinks that the subject of keeping children's teeth clean is of very great moment; on which point dentists ought to take great pains to instruct the public. Adjourned till 2 P. M.

### AFTERNOON SESSION.

The subject of children's teeth was resumed.

Dr. Clarke spoke at some length of the action of acids on the teeth; acids in food; acids in the secretions, and acids generated by foul deposits between and around the teeth.

Dr. Perkins believed that the luxuries of the present age in this country, are destroying teeth with alarming rapidity, by vitriolating the general constitution, as well as by direct action on the teeth.

Dr. Quinlan thinks that the experiments already made show clearly that acid substances injure the teeth.

Dr. Fitch thinks the great question is, what acids the food develops, and what action they have upon the teeth, and believing that the subject had been sufficiently discussed for the present, he suggested that the proper topic for the afternoon's discussion, is, the propriety of filling or plugging the fangs of teeth.

*Fang Filling.*—Dr. Clarke said that the filling of the fangs of teeth was practiced long before he was in dental practice. Drs. Harwood, Maynard and others, were before him in this department of the art of dentistry. His present confidence in *fang filling* is greater than he had ever anticipated.

The method of operating is to draw the temper from a fine broach and cut barbs like those of a fish-hook, covering one entire side, leaving the other side perfectly smooth. With the smooth side against the side of the orifice, he then thrusts the instrument to the bottom of the nerve cavity to the very foramen of the root; whereupon he quickly rotates the instrument so as to bring its barbed side in contact with the nerve, and then extracts it from the cavity whereby the nerve is removed. The cavity is farther cleaned with small instruments, when it is ready for the filling. In filling the cavity I use one instrument, the temper of which is drawn to the blue, and wrap around it a fold of gold foil, which I thrust to the bottom of the root, and press to one side of the cavity. The same operation is repeated till the entire nerve cavity is firmly filled with the metal.

If the disease is wholly eradicated, and the cavity entirely and faithfully filled, a great change takes place around the tooth immediately. But we should not promise our patients too much. Fang filling is uncertain. We do not cure the disease of the tooth; we merely remove obstructions to its well-being, and nature performs the cure.

Dr. Knapp said:—The removal of the pulp of the fang is sometimes difficult, also the filling of the fang, especially of the molars. Kreosote is the best agent for cleaning out the *pus* from the cavity.

Dr. Anderson uses kreosote and arsenic to cleanse a nerve cavity and prepare it for filling. The substances are introduced on cotton by means of a broach.

Dr. Kennicott frequently extracts the nerve by the use of a piece of hickory, and sometimes stops the cavity with a well fitted hickory plug.

Dr. McKellops uses nitrate of silver, 60 grains to the oz., to clear out the fang, and employs an instrument manufacted by the New York Teeth Manufacturing Company, which is excellent for the purpose.

Dr. Perkins uses the chloride of soda to inject the fangs of teeth. Sometimes the fang has an ulcer when there is no other disease in the tooth.

Dr. Clarke treats ulcerated fangs by thrusting kreosote up the orifice on cotton, and leaving it there from time to time for some days.

Dr. Dunham did not think favorably of the hickory plug spoken of by Dr. Kennicott.

Dr. Clarke stated that in cases where the fang of a molar with a lateral decay to the nerve, required to be stopped with gold, he was in the habit of drilling a hole through the grinding surface of the enamel, in order to get access with his broach to the orifice of the root.

At this stage of the proceedings, on motion of Dr. Allport, the following gentlemen, not residents within the geographical limits of the society, were elected as honorary members : Drs. S. Brown, of New York; Perkins, of Rome, N. Y., Smith, of Terre Haute, Ind.; Clarke, of New Orleans, La.; Knapp, of New Orleans, La.; Knapp, of Jackson Miss.

EVENING SESSION.

*Subject—Gutta Percha as a base for teeth.*

Dr. Dunham thinks gutta percha very useful in cases where the teeth have been recently extracted, and the alveolus very prominent, especially about the eye teeth. In these cases it is often very difficult to use metallic plates without making the lips too prominent. He prefers to use a thin plate of gutta percha rather than a gold plate, the ends of the teeth resting on the gum without a plate in front.

Dr. Smith has used gutta percha in two cases, but does not like it; especially the samples which he had, and which were said to have been made by Dr. Slayton. The texture and color were both bad.

Dr. Quinlan is of opinion that gutta percha will be found in many cases to be very useful.

Dr. Spaulding uses gold plate for temporary cases as the best material. When the gum changes much he uses gutta percha to fill up the interstice or chasm between the plate and the arch of the palate and the alveolar ridge. This plan he finds to work well.

Dr. Clarke does not use gutta percha for permanent sets of teeth, but nevertheless regards it as useful for many purposes, particularly when a piece of work constructed upon it brings the patient back in quick time for a new set on proper material. He occasionally uses it for patching up old work, which is generally neither very pleasant nor very profitable to the dentist. He thinks that when a gutta percha plate wears through very soon, it is owing to the material having been overheated, or burned by the lamp.

For lower pieces, where only the front teeth are gone, he likes gutta percha, yet he has no reliance upon it for any but temporary sets.

SECOND DAY

The Secretary read the minutes of yesterday, which were amended and adopted.

Dr. Clarke was requested by Dr. Spaulding to state whether solder made with arsenic, will eat up the platina plate.

Dr. Clarke thinks it will if the arsenic is in excess. The sanguine hopes at first expressed, have not been fully realized. The experiments have not thus far been fully satisfactory, but he hopes the difficulties will be ultimately overcome.

Dr. Honsinger had one job of continuous gum, which was not successful, and only one. He proceeded to describe the case minutely. A set of gum teeth had failed in the mouth. He substituted continuous gum. This soon lost one molar, and the gum was broken and loose. Made a new set, and that gave way. Then struck a second plate and added it to the first, bringing the edge over the front enamel. This also gave way, probably on account of the many bakings.

Dr. Clarke advised Dr. H. to send his subject to Dr. Harris, of Philadelphia, who once had a similar case, which he made of cast iron, the patient wearing it to the present time.

Dr. Spaulding said, in relation to the articulation of continuous gum work it must be good, but when the antagonizing teeth are gone, the danger of unequal pressure can hardly be avoided.

Dr. Perkins had encountered the same difficulty. In such cases he has made the backing and front part of the plate exceedingly strong, and put a block of platina over the place of strongest pressure.

Dr. McKellops has done a good deal of continuous gum work. One piece after six months, exhibited the four front teeth projecting forward. He split the bakings and turned one-half the back up on the plate; then baked carefully; merely soldered the backs to the plate, not the pivots.

Had a case where the patient had worn gold plate; first made a block—then substituted gum work. The principal strength of this work is in the baking; the first bake of the body should be but just sufficient to cause the material to adhere.

A young gentleman present was called on by the President to explain his method of making gum work. Described the process generally, but owing to the want of time, too briefly to be of much practical use.

Dr. Blake thinks that the baking should be *progressive*—each baking more than the former, fusing the whole mass.

Described a case of filling up the cheeks, which was fully successful, and is of opinion that continuous gum work is of considerable use to the profession.

Dr. Clarke says, of air chambers, that sometimes they are best, but not always. When the arch of the mouth has a hard protuberance, an air chamber is necessary; but not when the arch is spongy. He makes a large air chamber in the former case. Sometimes has difficulty with both kinds of plate.

In regard to articulation, the best teeth for use are those where the bicuspids are pitched inward. But you cannot follow the natural laws, in articulating artificial teeth. In teeth set by our best operators, the *circle* is more perfect than in the natural teeth.

Dr. Dunham is pleased with the character of the discussion this morning. Is obliged to Dr. Honsinger for his particular delineation of his *failures*. This is even more useful to us than a description of our *successes*.

Dr. Dunham sometimes uses air chambers in the cases stated by Dr. Clarke, and does not use them in soft spongy gums.

Describes a plan of making air chambers. Cuts a piece of tin an inch long and a half or three-quarter inch wide; after swaging his plate he places the tin in the female cast and strikes the plate over it. This prevents the rocking.

He takes upper impressions generally in wax; lower ones in plaster.

When the wax is introduced, he directs his patient to press his fingers against the cheek and wax over the molars, whilst he presses up the cup to its position. He presses the front wax a little inward, after taken from the mouth.

Dr. Bogue wished the opinion of gentlemen as to air chambers.

Dr. Dunham generally takes three casts, and selects the poorest to swage on at first, then if the fit is bad, on another, and so on to the last, if necessary.

Dr. Spaulding uses air chambers when the hard prominence exists in the arch. In almost all cases of partial sets he uses air chambers.

Makes the edge of the chamber square and not rounded, to prevent the expansion of muscle into the chamber.

In order to produce the square edge he cuts out a piece of the thin plate. Though shallow, the chamber will not fill with flesh; and its impression is scarcely perceptible to the eye Considers them better than deep chambers.

On the subject of impressions he has settled upon wax exclusively unmixed.

Uses sheet zinc to strike off a preparatory plate or cup. In this plate he places sheet wax of equal thickness and warmth. When the gum is spongy he cools the wax a little.

In lower plates when the sheet of wax is placed on the zinc plate, he gently presses the wax on the zinc die, to give an *approximation* to the true forms. Takes cold water into the mouth to cool the wax.

In lower plates it was his custom to limit the plate at the movable muscles. Draws a line on his plaster with a pencil where he judges the muscle will move. Increases the suction by remodeling the edge of the plate in front.

One of the most difficult points to fit is behind and around the condyle of the jaw. Attaches the rim to the edge of this curved edge of the plate. This curved edge not only increases the suction, but may be made to fill up the mouth and keep out the lip as desired. Around the condyle it is sometimes necessary to cut out a wedge of the plate. Sometimes injects cold water about the condyle to harden the wax before removing it.

The zinc plate may be quarter of an inch wider than the gold plate. Does not spread the zinc plate at all at the sides.

Dr. Dunham asks whether Dr. Spaulding ever springs the zinc plate by unequal pressure. The body of wax must be as thin as possible, and not break, also the wax must be very soft.

A hole must be in the centre of the plate to let in the air before removing.

Sometimes he pares off a little of the plaster cast in front, to compensate for the imperfect fit over the edge of the alveolus.

Dr. Smith, of Ottawa, wished to know whether an *atmospheric pressure plate* is the same as a *suction plate.*

Dr. Dunham differed from Dr. Spaulding on the subject of atmospheric pressure ; he believes that atmospheric pressure does exist, because if a small orifice be made into an air chamber the plate will drop.

Dr. McKellops must say of central cavities that they are useful. Thinks that in flabby mouths plaster is better than wax.

Dr. Spaulding responds that wax impressions caused no more necessary pressure than plaster, if the wax is sufficiently warm.

Engrave a groove in the exact place on the plaster cast where the plate is to terminate on the palate. This forms a flange on the plate for its more perfect adaptation.

Dr. Anderson has thought the air chamber was sometimes used for ornament. Thinks the prettiest form of a plate is that of nature. Thinks the pressure should be removed from the palatial arch for the purpose of avoiding the rocking of the plate. For this purpose he removes an equal surface of the plaster cast, excepting near the posterior part. This plate will ultimately settle about enough to fill up the cavity. Thus the pressure is brought upon the alveolus where it belongs.

Dr. Clarke says, we have two principles, viz. *cohesion* and *atmospheric pressure.*

Capillary attraction is a very different matter; it pertains to fluids and small tubes, like those in the sponge.

Dr. Fitch denies that cohesion or attraction has anything to do with the plates of teeth. They adhere from atmospheric pressure. Has found air chambers in some cases important.

Dr. Spaulding thinks the perfect fit of the margins of the plate more important than the fit in its middle parts.

Dr. Perkins denies cohesive attraction and asserts atmospheric pressure, in the case of plates.

Professor Palmer, of the Michigan Medical University, was requested to explain the terms cohesion and adhesion, which he did.

Two kinds of mechanical attraction—all matter in creation has the attraction of gravity, operating at all distances. Also an attraction when brought into absolute contact, called cohesion. He says there is no essential difference between cohesion and adhesion.

Thinks the *adhesion* by the atmosphere is not identical with the *cohesion* of the books.

Adjourned till half-past 2 P. M.

### SECOND DAY—AFTERNOON SESSION.

Dr. McKellops moved that the constitution of this Society be published.

*Resolved*, That the Secretary be requested to furnish Dr. S. Brown with a copy of the constitution for publication in the Forcep.

The subject of gold as fillings for the teeth, was the order of business.

Dr. Clarke has found that crystal gold has already far surpassed his expectations. He has seen several teeth filled by Dr. Allport, of Chicago, that surpass anything he had ever before seen.

An example of two central incisors from which the ends of the teeth had been removed one-fourth of its length. These plugs have now stood about 19 months, and are as perfect as at first. He has never seen anything so fine.

This class of operations has of late done more to elevate the profession than anything else. The class of men who are doing this kind of work encourage us all to make high endeavors in our art.

In thus complimenting Dr. Allport, he does not mean to say that he is going home to throw away his tools, and turn blacksmith. No such thing, but he is going back to endeavor to imitate these great examples.

Dr. Smith once thought he could plug a tooth tolerably well. But he had lately seen a first bicuspid, filled by Dr. Allport, two-thirds of which above the gum was of crystal gold. It preserved the original form of the tooth and performed all the purposes of mastication.

Dr. Knapp (of N. O.) thinks that cylinders are the best form of gold for plugs as a general thing. This system of cylinders used by Dr. Clarke, he thinks is new. That any former methods claimed to be such were quite different from this. Crystal gold will doubtless succeed in some cases, especially in building up parts of teeth, such as he had seen of Dr. Allport's, better than any kind of foil.

Dr. Knapp, of Miss., was here named and elected an honorary member of the Society.

He returned thanks for the honors, and stated that he has succeeded better with cylinder fillings than with gold in any other form.

Dr. Spaulding stated that he had regarded crystal gold as the very best material for filling very shallow cavities; and also in building up structure, the crystal gold is the perfection of material.

Cylinders will make the best filling in the shortest period of time, and with sure labor.

The first specimen of cylinder stoppings I had seen were by Dr. O. P. Laird, of Columbus, Ga., when he was in Savannah. He saw also ten years ago plugs by Dr. Badger, which were very excellent, better than himself can do. He had understood that Dr. Badger used cylinder plugs. He formerly suggested to Dr. Clarke the propriety of seeing Dr. Badger—but Dr. Clarke has made improvements in the method of forming cylinders. He does not wish to deprive any gentleman of his laurels.

On the whole he regards cylinder plugs as equal to any other, not excepting crystal gold. The plugs of ordinary form, of crystal and cylinders respectively, are so nearly equal that the difference is not worth regarding.

Dr. Clarke has always aimed to accord to another dentist all his rights and improvements. In the winter of '49, in New Orleans, he formed the acquaintance of Dr. *Badger*, and was perfectly satisfied that Dr. B. could excel him in filling teeth.

He gave me a hint of his method, by rolling a piece of leaf into a cylindrical form. I then entered upon my plan of rolling cylinders on small tweezers, such as I had seen in the hands of Dr. Badger, which he used for another purpose.

I have stated often that I received my first idea of cylindrical fillings from Dr. Badger, I have claimed nothing for myself. Whatever I have belongs to the profession.

Dr. Allport thinks the profession are pretty well aware who is entitled to the credit of inventing this method of filling teeth, and whoever he is, he enjoys the satisfaction of knowing it.

Dr. Knapp (younger) says there is nothing before this body to show that Dr. Laird ten years ago filled teeth with cylinders.

Dr. Kennicott says that as long ago as 1820 he saw Dr. Cox in New Orleans fill teeth with cylinders.

He has failed with crystal gold, many times. Not a particle of moisture must touch a gold filling of any kind.

Dr. Honsinger thinks that crystal gold has mostly failed in consequence of having been introduced in too large quantities. He uses very small instruments. Has built up teeth when one-third gone.

Dr. Bogue gives his testimony decidedly in favor of crystal gold.

Dr. Clarke has used but little crystal gold, but wishes to know whether the serrated instruments do not fracture the surface of the cavity, in consequence of their sharpness.

Dr. Perkins—I have some experience in this department of filling with cylinders.

If any persons will come to Rome, I will show them fillings built up with foil by Dr. Allport eight years ago, in fine condition now.

I have used Watt's gold from the commencement of his manufacture, and regard it a very useful adjunct to foil. Has recently used about one-third crystal gold.

Does not think a person ought to say that foil cannot be made to perform anything that crystal gold can do. Nor can he assert positively that it can.

He described at length the manner of stopping with crystal gold.

Dr. Clarke requested that Dr. Allport would describe his method of stopping with crystal gold.

Dr. Allport could hardly express his gratitude for the kind and flattering compliments which had been bestowed upon him.

Does not use crystal gold exclusively. Foil constitutes about two-thirds of his fillings. Sometimes he uses pellets, and cylinders and crystals all in the same cavity. The use of crystal gold requires four times the time for insertion that is required by foil.

Never saves any more of the enamel than is strong and healthy—but saves all the front possible. He presses the crystals in the direction of the length of the tooth as much as possible. Uses serrated instruments, small and sharp.

If a plug gets wet when of foil or pellets, or crystals, it can be cleaned by cloroform and wiped dry with bibulous paper.

The credit due to crystal gold is attributable to Dr. Watts. Since July last his gold has been good, very good—all that is required.

He can build upon a surface after it has been polished, or upon solid metal.

Dr. Spaulding explained the reason why serrated instruments packed the crystals in such a manner as to cause them to cohere in a mass, but it must be borne in mind that the serrated points do not push all parts of the crystals with equal solidity, at the polished surface they become nearly so.

Dr. Dunham does not think he could stop front teeth better with crystals than with foil; he never allows the force to fall wholly upon one tooth, but introduces a wedge between the teeth, to connect the force with two or more teeth.

The Society next proceeded to the consideration of

### CONTINUOUS GUM WORK.

Dr. Spaulding thinks the gum and body may be made strong enough.

Dr. Kennicott gives his word for continuous gum work and esteems it the perfection of artificial teeth.

Dr. Clarke is using it pretty extensively, and thinks it occupies an important place in the profession. Deems it as useful as any kind of work that has ever been made. It is artistic in an eminent degree, and is not liable to the objections raised against gutta percha, that it lowers the standard of professional skill.

Dr. Spaulding assents to all that Dr. Clarke has said in favor of continuous gum, yet there is one serious objection, which is the liability of breaking the teeth either in the process of manufacture, or by a fall afterwards, in which case it is a sort of work very difficult to repair.

Dr. Dunham regards the breaking of the gum as a much greater objection than the breaking of the teeth. This he has not been able to avoid.

Dr. Kennicott has seen gum work that nothing else has ever equalled. It can be adapted in color both of gum and teeth to suit the complexion. The frequent heating of this work is very liable to injure the teeth.

Dr. Perkins regarded it as unfit in all cases.

Dr. Smith desired to know whether tobacco did not make gutta percha sets very disagreeable. Dr. Clarke replied that a smoker would find his teeth disagreeable.

Dr. Dunham has charged $75 for sets of gutta percha teeth, which is the regular price in St. Louis. Thinks twenty-five of his patients are wearing them comfortably.

Dr. Allport has used it in cases where it answered a very good purpose for temporary work.

Dr. McKellops moved that when the Society adjourn it be to meet in the city of St. Louis, on the 3d Wednesday in May next. Adjourned.

In the evening of the 31st an entertainment was given by the dentists of Chicago at the Briggs Hotel. In addition to the many entertaining and instructive speeches by the members of the dental profession, Dr. Palmer, one of the professors of the Michigan Medical University, who knows exactly what to say, with decided ability to say what he thinks and wishes in the most fluent and condensed phraseology, made two eloquent addresses, with which the members of the Society and invited guests were greatly delighted.

The Chicago meeting of the Dental Society of the West will long be remembred with pleasure and profit by those present.

Solyman Brown.

[*Forceps.*

